# Errata

**Published:** 1785

**Authors:** 


					p 22x, 1, a3, after Frankreicby add befondcrs ubtr
c\e fpitaltr.?

				

